# NIR-Mediated Deformation from a CNT-Based Bilayer Hydrogel

**DOI:** 10.3390/polym16081152

**Published:** 2024-04-19

**Authors:** Shijun Long, Chang Liu, Han Ren, Yali Hu, Chao Chen, Yiwan Huang, Xuefeng Li

**Affiliations:** 1 Hubei Provincial Key Laboratory of Green Materials for Light Industry, Hubei University of Technology, Wuhan 430068, China; liuchang9708@163.com (C.L.); renhann@126.com (H.R.); hu_yali1@163.com (Y.H.); yiwanhuang@hbut.edu.cn (Y.H.); 2 Hubei Longzhong Laboratory, Xiangyang 441000, China; 3 New Materials and Green Manufacturing Talent Introduction and Innovation Demonstration Base, Hubei University of Technology, Wuhan 430068, China; 4 Hubei Key Laboratory of Polymer Materials, Hubei University, Wuhan 430062, China; chenchao@hubu.edu.cn

**Keywords:** temperature-responsive hydrogel, particle double-network, photothermal conversion, actuator, carbon nanotubes

## Abstract

Shape-shifting polymers are widely used in various fields such as intelligent switches, soft robots and sensors, which require both multiple stimulus-response functions and qualified mechanical strength. In this study, a novel near-infrared-light (NIR)-responsible shape-shifting hydrogel system was designed and fabricated through embedding vinylsilane-modified carbon nanotubes (CNTs) into particle double-network (P-DN) hydrogels by micellar copolymerisation. The dispersed brittle Poly(sodium 2-acrylamido-2-methylpropane-1-sulfonate) (PNaAMPS) network of the microgels can serve as sacrificial bonds to toughen the hydrogels, and the CNTs endow it with NIR photothermal conversion ability. The results show that the CNTs embedded in the P-DN hydrogels present excellent mechanical strength, i.e., a fracture strength of 312 kPa and a fracture strain of 357%. Moreover, an asymmetric bilayer hydrogel, where the active layer contains CNTs, can achieve 0°–110° bending deformation within 10 min under NIR irradiation and can realise complex deformation movement. This study provides a theoretical and experimental basis for the design and manufacture of photoresponsive soft actuators.

## 1. Introduction

Stimuli-responsive hydrogels, also known as smart hydrogels, are materials that respond to stimuli in the external environment and undergo considerable changes in colour [1,2,3], volume [4] and mechanical properties [5]. According to different stimulus-response modes, they can be roughly divided into temperature-responsive hydrogels [6], electrical-responsive hydrogels [7], light-responsive hydrogels [8,9], magnetic-responsive hydrogels [10,11,12] and chemical-responsive hydrogels [13,14]. Because of these characteristics, they have various applications in intelligent switches [15,16], artificial muscles [17,18], soft robots [19], shape-memory materials and other fields [20,21,22,23]. The use of temperature-responsive hydrogels is a relatively mature and simple method. It is a type of hydrogel material that can produce phase changes in the process of temperature change, usually with a lower critical transition temperature (LCST) or an upper critical transition temperature. As a typical representative of a temperature-responsive hydrogel, Poly (N-isopropyl acrylamide) (PNIPAM) hydrogel has attracted much attention because of its LCST (32 °C), which is close to human body temperature [24]. At temperatures below the LCST, the polymer chains of PNIPAM tend to form hydrogen bonds with water molecules, which are in a stretched state, causing the hydrogel to swell. At temperatures above the LCST, the polymer chains of PNIPAM are prone to forming hydrogen bonds on their own, expelling water and causing the polymer network to collapse. However, NIPAM hydrogels only have a single function; therefore, they have a narrow application range and are often restricted in complex environments. To overcome this, a feasible strategy is to use multiple functional substances to achieve the multiple stimulus responsiveness of hydrogels.

By contrast, light, as a noncontact and remotely controllable stimulus, can switch between two-dimensional and three-dimensional spatial scales and can be easily controlled in the temporal dimension by turning on and off the light source. In addition, light energy is adjusted by its own properties (e.g., wavelength and intensity), commonly including near-infrared (NIR), visible (vis) and ultraviolet light [25,26,27]. Light stimulation can change the physical and chemical properties of a hydrogel, primarily by changing its network structure. According to the action mechanism, the effects of light stimulation on hydrogels can be divided into three categories. First, light can directly cause the formation and breaking of chemical bonds and change the cross-link density of hydrogels. Moreover, light of a specific wavelength can isomerise corresponding photosensitive molecules, such as the cis/trans isomerisation of azobenzene and the open and closed loops of spiropyran, which can cause sol–gel transitions or change the swelling performance of the hydrogel. In addition, nanomaterials with photothermal properties, such as carbon nanotubes (CNTs) and graphene, can be introduced into hydrogel systems. These materials absorb light energy and convert it into thermal energy, thereby triggering thermally responsive hydrogels.

Herein, we modified CNTs using a silane coupling agent, introduced reactive groups to the surface of the CNTs and prepared CNT composite hydrogels. Because of the existence of NIPAM and CNTs, hydrogels can respond to thermal and light stimuli. Combined with a particle double-network (P-DN) hydrogel strategy, it also has good mechanical properties. Furthermore, we prepared a hydrogel actuator using a photothermal conversion function with an asymmetric bilateral structural design, which can realise rapid bending deformation under infrared light stimulation.

## 2. Materials and Methods

### 2.1. Materials

N-isopropyl acrylamide (NIPAM, 98%) and methyl acrylate (MA, 98.5%) were supplied by Shanghai Macklin Biochemical Co., Ltd., Shanghai, China. Hydroxylated multiwalled carbon nanotubes (95%), triethoxyvinylsilane (TEVS, 97%) and N,N,N′,N′-tetramethylethylenediamine (TEMED, 99%) were supplied by Shanghai Aladdin Bio-Chem Technology Co., Ltd., Shanghai, China. Ethanol absolute (analytical reagent), benzene (analytical reagent), Tween 80 (chemically pure), ammonium persulfate (APS, analytical reagent) and N,N′-methylene diacrylamide (MBAA, chemically pure) were supplied by Sinopharm Chemical Reagent Co., Ltd., Shanghai, China. Poly(sodium 2-acrylamido-2-methylpropane-1-sulfonate) (PNaAMPS) was prepared according to the method used in our previous study [28].

### 2.2. Modification of CNTs

The silane coupling agent (triethoxyvinylsilane) is entangled and coated on the surface of the CNTs after refluxing, hydrolysis and condensation in water. The experimental process is as follows. Hydroxylated multiwalled CNTs (1.0 g) were added to deionised water (200 mL) in a beaker, which was then placed in an ultrasonic cell breaker and sonicated for 30 min under ice-water bath conditions at 270 W. After adding triethoxyvinylsilane (5.65 g) and anhydrous ethanol (40 mL), the mixture was placed in the ultrasonic cell breaker and sonicated. After ultrasonic dispersion, the CNT diversions were poured into a 500 mL three-necked flask in an oil bath at 88 °C for 24 h. The speed of the mechanical stirrer was adjusted to 280 rpm. After the reaction, the product was filtered using a Buchner funnel. The reaction product was first rinsed with a large amount of benzene and then with a large amount of ethanol. When no liquid droplets were visible on the surface of the CNT mixture, the product was placed onto aluminium foil and dried in a vacuum drying oven at 100 °C for 24 h.

### 2.3. Preparation of the Hydrogel

The photothermal PNaAMPS/P(NIPAM-co-MA)/CNT hydrogels were prepared from a precursor aqueous solution containing 2.0 mol L^−1^ NIPAM, 10 wt% hydrophobic monomer MA, Tween 80 emulsifier (1.0 wt% of water), the photothermal component CNTs (0.00–0.20 wt% of the total monomer), 0.1 mol% of the chemical cross-linker MBAA and 0.1 mol% redox initiators. Initiators (APS and TEMED) were added to the solution at room temperature to form a mixed aqueous solution. The solution was transferred into a reaction cell (100 mm × 100 mm) comprising a pair of parallel glass plates and polyester films separated by a hollow silicone rubber spacer ≈ 1 mm thick. The solution was polymerised in a refrigerator at 5 °C for 12 h to form the PNaAMPS/P(NIPAM-co-MA)/CNT hydrogel.

### 2.4. Fabrication of the Bilayer Hydrogel

The upper hydrogel layer (active layer) containing the CNTs was prepared according to the method described in Section 2.3. After polymerisation, the prepolymer solution of the second hydrogel layer (negative layer) was added to the first hydrogel layer. The second layer of the hydrogel prepolymer solution did not contain CNTs, and the remaining preparation steps were identical to those used for the first layer described in Section 2.3.

### 2.5. Characterisation of Hydrogels

#### 2.5.1. Fourier Transform Infrared Spectrometry

The samples were dried in a 90 °C vacuum drying oven for 24 h and then ground with an appropriate amount of potassium bromide powder to obtain a fine and uniform powder. Before testing, the sample and potassium bromide powder were placed in a 90 °C vacuum drying oven for 30 min. Fourier transform infrared spectrometry (Tensor II; Bruker Co., Ltd., Karlsruhe, Germany) was undertaken between 4000 and 500 cm^−1^.

#### 2.5.2. Raman Spectrometry

The low-frequency analysis of gel samples was performed using a microconfocal Raman spectrometer (XploRA PLUS; HORIBA France SAS., Palaiseau, France) with a 532 nm argon ion laser as the light source. The Raman spectroscopy measurements were conducted at room temperature with a displacement range of 200–3000 cm^−1^.

#### 2.5.3. Field-Emission Scanning Electron Microscopy

The microstructure of the hydrogel was observed using field-emission scanning electron microscopy (FESEM; SU8010; Hitachi Co., Ltd., Hitachi, Japan). Specifically, the sample was frozen and fractured in liquid nitrogen, followed by freeze-drying for 48 h. Before the FESEM characterisation, a thin layer of gold was applied to the fractured surface of the sample by sputtering. The acceleration voltage of the FESEM was 5.0 kV.

#### 2.5.4. Tensile Measurements

At room temperature, uniaxial tensile tests were performed with rectangular strips (30 mm × 5 mm) of the samples using a universal tensile testing machine (CMT6103; MTS Co., Ltd., Shanghai, China) equipped with a 1 kN load cell at a constant stretching velocity of 100 mm min^−1^. The elastic modulus *E* was calculated from the slope of the initial linear region of the stress–strain curve (within the range of 5–10%). The extension work was calculated from the area under the stress–strain curve to the fracture of the uncut sample. The nominal stress *σ* was calculated from the tensile force and the initial cross-sectional area of the undeformed sample. The strain rate *ε* was defined as the ratio of the stretching speed to the original gauge length. Unless otherwise specified, three measurements were performed for each sample.

#### 2.5.5. X-ray Photoelectron Spectrometry

To accurately analyse the chemical composition and elemental chemical state of the sample, an X-ray photoelectron spectrometer (PHI-5000 VP-III; ULVAC, Inc., Chigasaki, Japan) was used in the experiment. We used a monochromatic X-ray, K-α aluminium microfocusing light source and 0.60 eV photon energy. The sputtering source formed a grating on the surface of the sample with a rectangle of 1.40 mm × 1.40 mm

#### 2.5.6. Equilibrium Swelling Ratio

The swelling properties of the hydrogels were measured over a wide temperature range. First, the hydrogel samples were placed in deionised water and maintained under various temperatures to reach the swelling equilibrium. They were then removed from the deionised water and their surfaces were wiped with filter paper before measuring their weight (*W_s_*). Finally, the samples were fully dried in an oven at 100 °C to determine their weight after drying (*W_d_*). The equilibrium swelling ratio (*ESR*) was determined as follows:

$$ESR\left(wt\%\right)=\frac{W_{s}-W_{d}}{W_{d}}\times 100\%.$$

#### 2.5.7. Photothermal Conversion

The swelling-equilibrated hydrogel samples were cut into discs with 1 cm diameters, placed on glass slides and irradiated with a 980 nm infrared laser maintained at 2.0 W. The temperature changes in the samples were measured using an infrared camera (C5; FLIR Systems, Inc., Wilsonville, OR, USA). The infrared camera was set to infrared thermal imaging mode, and the sample was photographed at a distance of about 0.5 m above. When the temperature rose rapidly during the first minute, the image was recorded per 10 s. After the temperature gradually stabilised, it was recorded per 30 s.

#### 2.5.8. Three-Point Bending Test

At room temperature, three-point bending tests were performed with rectangular strips (30 mm × 10 mm) of the samples using a universal testing machine (CMT6103; MTS Co., Ltd., Shanghai, China) equipped with a 1 kN load cell at a constant stretching velocity of 10 mm min^−1^. The span was set as 15 mm. The flexural modulus *E* was calculated from the slope of the initial linear region of the stress–strain curve (within the range of 5–10%).

## 3. Results and Discussion

**Scheme 1:**
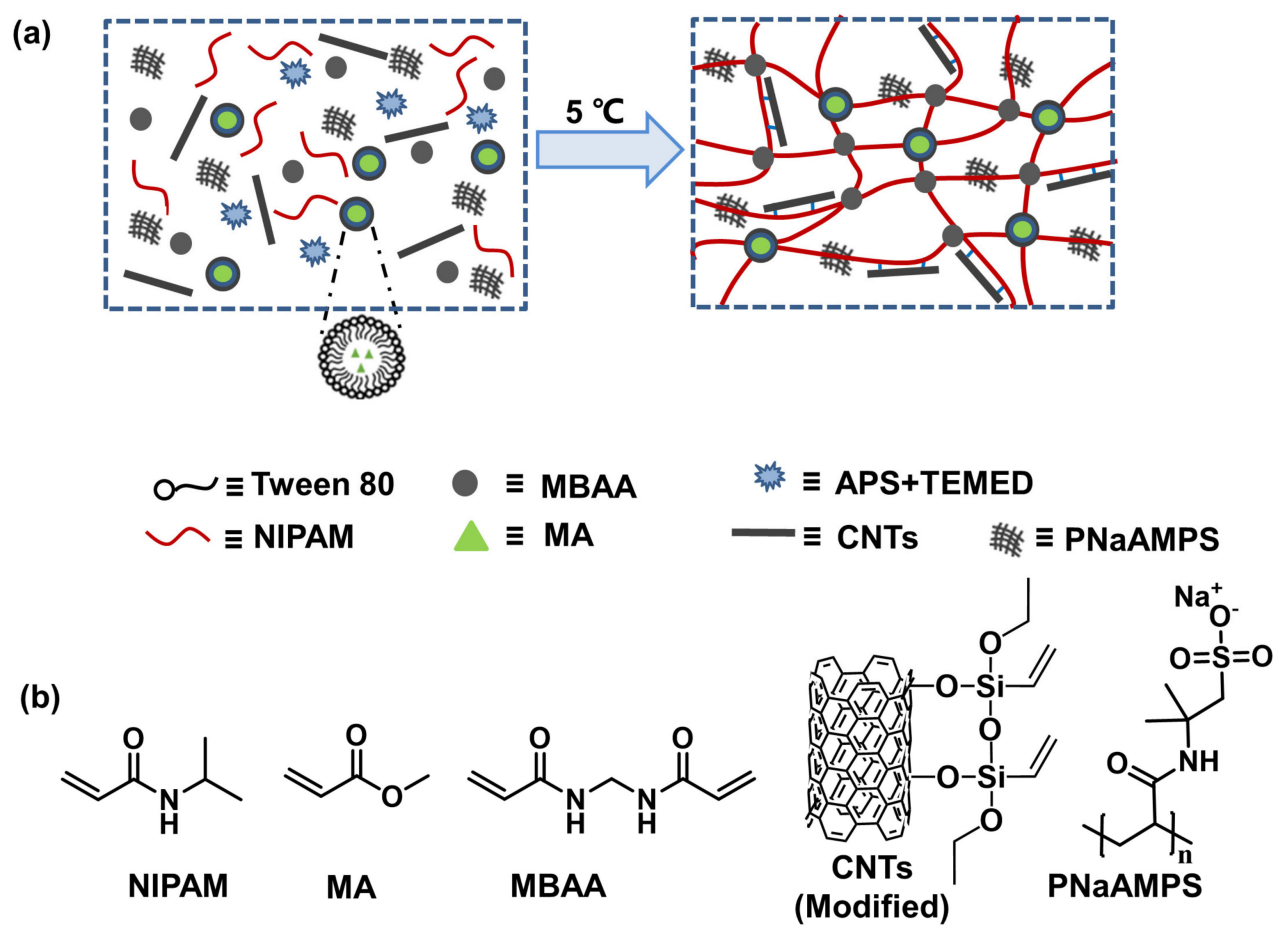
Preparation of the PNaAMPS/P(NIPAM-co-MA)/CNT hydrogel. (**a**) Hydrogel synthesis mechanism and (**b**) the structural formula of the main components.

Here, we report on the fabrication of a hydrogel with photothermal conversion and deformation abilities. As shown in Scheme 1, the hydrogel contains NIPAM, MA, PNaAMPS, MBAA, Tween80, APS, TEMED and modified CNTs. Each of these has the following different functions in the gel: (i) NIPAM and MA polymerise to form a continuous phase, while the PNaAMPS microgel acts as a dispersion phase, forming a particle double-network (P-DN) structure and dispersing in the hydrogel matrix; (ii) the MBAA is the cross-linker; (iii) the APS and TEMED are the redox initiation systems; (iv) Tween 80 is an emulsifier. In this hydrogel matrix, CNTs conduct photothermal conversion and provide mechanical enhancement. As a thermal-responsive component, NIPAM can synergistically act with CNTs to achieve photoresponsive deformation. Photothermal agent carbon nanotubes convert light energy into heat energy, and the PNIPAM component in P-DN hydrogel undergoes phase transformation and contraction when the temperature rises to the LCST. To add the CNTs to the hydrogel matrix to achieve an effective and stable connection, we used a silane coupling agent to modify the surface of the carbon nanotubes, introducing double bonds on its surface to achieve covalent bonding.

### 3.1. Characterisation of the Microstructure (Infrared Spectrum, Raman Spectrum, X-ray Photoelectron Spectroscopy and SEM)

**Figure 1:**
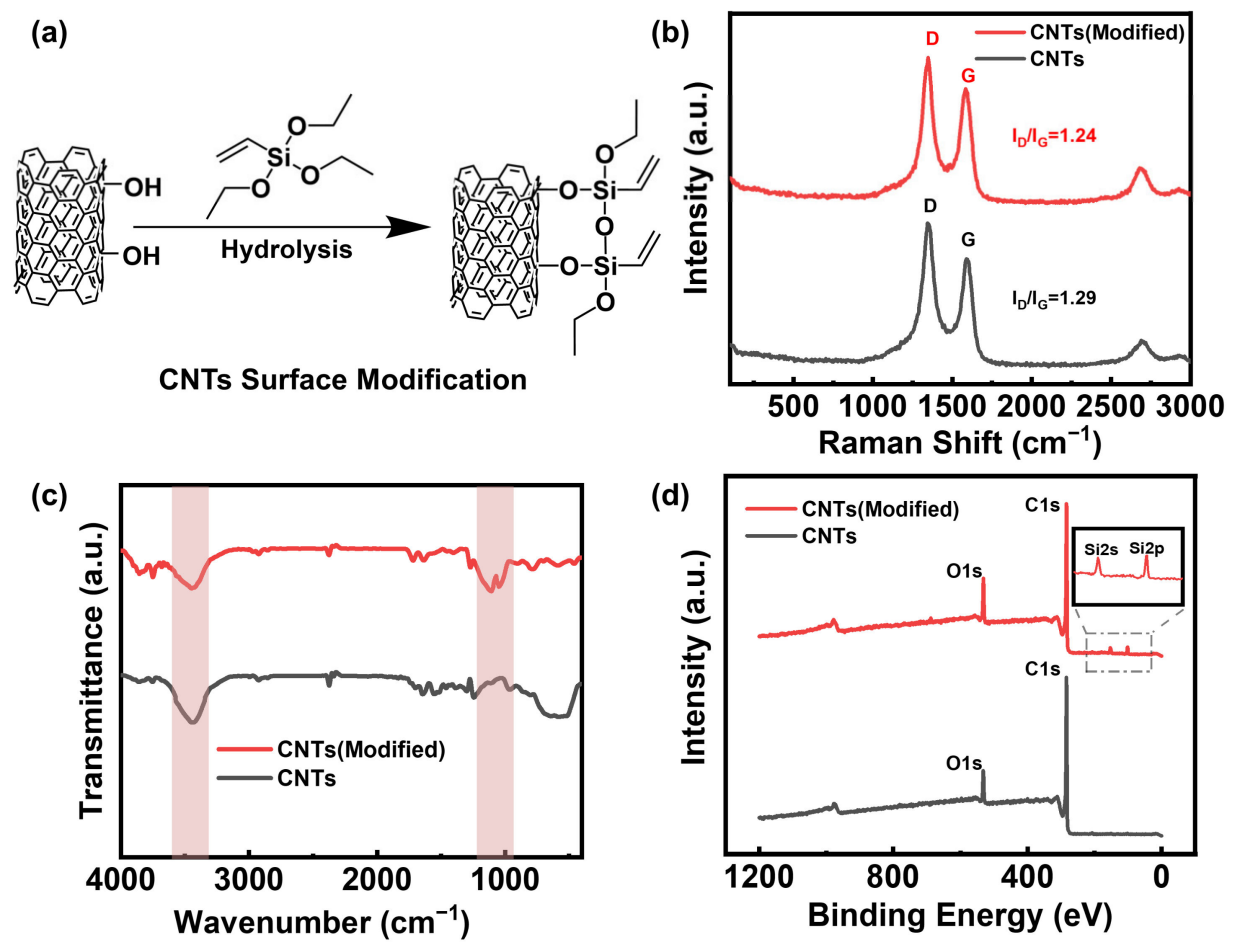
Microstructure characterisation of carbon nanotubes (CNTs) and modified CNTs. (**a**) Reaction principle of the silane coupling agent and the CNTs; (**b**) Raman spectroscopy of the CNTs and modified CNTs; (**c**) infrared spectroscopy of the CNTs and modified CNTs; (**d**) X-ray photoelectron spectroscopy of the CNTs and modified CNTs.

Figure 1a is a schematic of the surface modification of the CNTs using a silane coupling agent (triethoxyvinylsilane). Through the SEM images (Figure S1), we could not detect obvious surface morphology changes between the original and modified CNTs. According to the Raman spectra in Figure 1b, before and after modification of the CNTs, the G peak at 1590 cm^−1^ represents the six-membered carbon ring structure of the CNTs and the D peak at 1350 cm^−1^ represents defects in the structure. Therefore, the ratio of the intensity of the D peak to that of the G peak reflects the regularity of the six-membered carbon ring structure of the CNTs. Figure 1b shows that the six-membered carbon ring structure on the surface of the CNTs remains unchanged before and after modification, and the ratio of the D peak to the G peak remains largely unchanged. This is because the grafting reaction occurs on the CNT substituents rather than on the six-membered carbon rings. The infrared spectrum in Figure 1c shows that the hydroxyl absorption peak at 3440 cm^−1^ is weakened after modification by the silane coupling agent. This can be attributed to the decrease in hydroxyl groups on the surface of the CNTs after the silane coupling reaction. Simultaneously, characteristic Si–O–Si symmetric and antisymmetric stretching vibration peaks appear at 1032 cm^−1^ and 1106 cm^−1^, respectively, indicating that the silane coupling agent successfully grafted onto the surfaces of CNTs. The X-ray photoelectron spectroscopy spectrum in Figure 1d shows that, during the modification process, the oxygen content in the CNT sample slightly increases, which is speculated to be caused by the introduction of the silane coupling agent’s oxygen elements. In addition, after modification, the characteristic peak corresponding to the 2s and 2p orbital of Si appears (153 eV and 101 eV), indicating that the silane coupling agent was successfully branched onto the surface of the CNTs. From Table S1, we can observe that the silicon content increased from 0.00% to 4.44%, the oxygen content increased from 7.21% to 9.32% and, correspondingly, the carbon content slightly decreased from 92.69% to 86.24%. This can be attributed to the oxygen and silicon elements in the silane coupling agent reaching the surface of the carbon nanotubes, leading to an increase in the content.

Similarly, we also observed the characteristic peak of the Si-O-Si structure in the silane coupling agent in the infrared spectrum of the final PNaAMPS/P(NIPAM-co- MA)/CNT hydrogel, and also observed the silicon element in the XPS spectrum, so it can be judged that the target hydrogel has been successfully synthesised (Figure S2).

**Figure 2:**
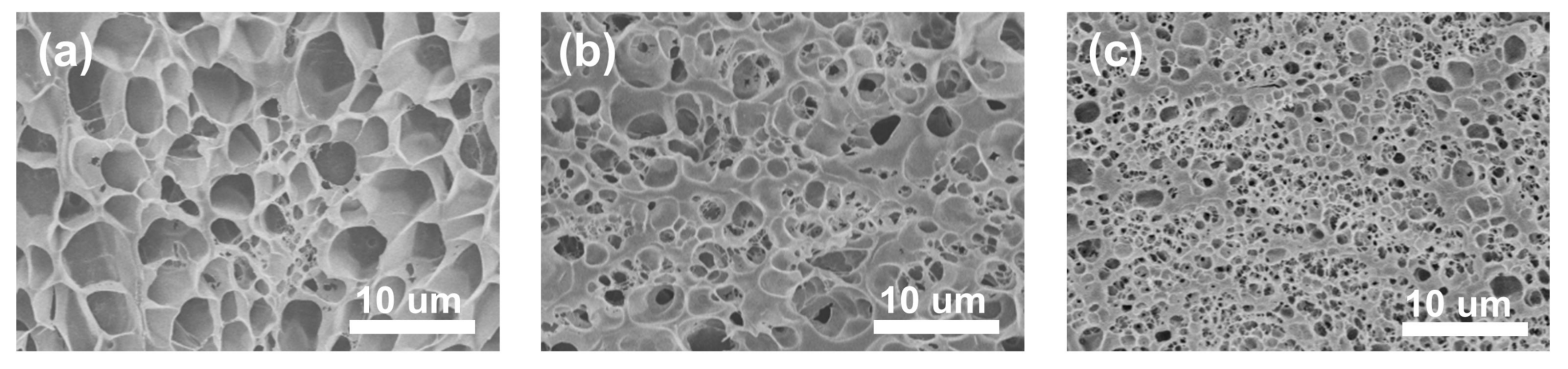
Scanning electron microscopy images of the hydrogels. (**a**) P(NIPAM-co-MA), (**b**) PNaAMPS/P(NIPAM-co-MA) and (**c**) PNaAMPS/P(NIPAM-co-MA)/CNTs.

Micromorphology analysis can explain the dispersion of the nanofillers in the nanocomposite hydrogels and the interaction between the filler and the matrix interface. As shown in Figure 2, the cross-sectional morphologies of the above hydrogels are typical porous structures and the network of the single-network gel is relatively loose, with large holes. Observed under the same magnification, compared with the single-network P(NIPAM-co-MA) hydrogel, the pores of the P-DN hydrogel are smaller and denser, which can be attributed to the denser polymer chain of the P-DN hydrogel. However, after the addition of CNTs to the double-network hydrogel, the polymer network becomes denser and the pore size gradually decreases, as seen in the SEM images in Figure 2. The possible reason is that carbon nanotubes are hydrophobic, which causes some of the water molecules to leave the hydrogel matrix, resulting in a denser hydrogel network.

### 3.2. Tensile and Swelling Properties

**Figure 3:**
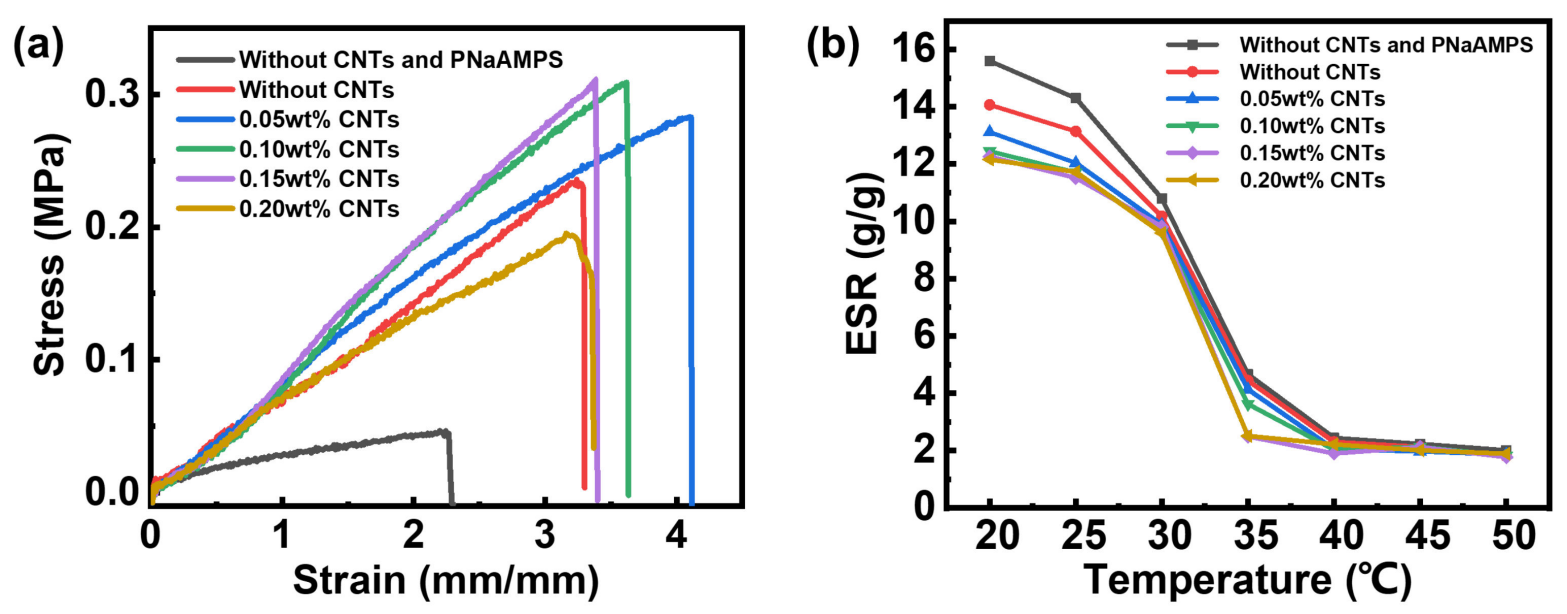
Mechanical and swelling properties of hydrogels with PNaAMPS/P(NIPAM-co-MA)/CNTs, PNaAMPS/P(NIPAM-co-MA) and P(NIPAM-co-MA). (**a**) Stress–strain curves and (**b**) equilibrium swelling ratio–temperature curves.

According to the stress–strain curve in Figure 3a, the mechanical properties of the P-DN hydrogel with the added PNaAMPS have been considerably improved, and the fracture strength, fracture strain and Young’s modulus have also been considerably improved. According to Table 1, as a control group, the fracture strength of the P(NIPAM-co-MA) single-network hydrogel without the CNTs was only 45 kPa, the fracture strain was 224% and the Young’s modulus was 40 kPa, reflecting the poor mechanical properties. The fracture strength, fracture strain and Young’s modulus of the P-DN hydrogel with the added PNaAMPS increased to 236 kPa, 324% and 47 kPa, respectively. This is attributed to the dispersion phase of the brittle PNaAMPS microgel breaking first, during which the network absorbed a large amount of energy and acted as a sacrificial bond, delaying the overall tensile fracture of the material [29,30]. Moreover, after adding a small number of CNTs to the P-DN hydrogel, the mechanical properties of the hydrogel improved slightly. When the amount of CNTs added was 0.15 wt%, the breaking strength, breaking elongation and Young’s modulus reached 312 kPa, 335% and 79 kPa, respectively, which increased by 32.20%, 3.40% and 68.09% compared with the hydrogel without the CNTs. As the concentration of the CNTs further increased, the tensile properties of the sample began to decrease. This is because, when the content is low, the CNTs have a fibre-strengthening effect on the hydrogel matrix. When the content is increased, the CNTs are clustered together and are difficult to disperse evenly; therefore, they form defects in the matrix. As a control group, the hydrogels added with the original CNTs reflected the poor mechanical properties (Figure S3), which failed to enhance the hydrogel. Such a phenomenon may be caused by the reduced dispersion of the unmodified CNTs.

Figure 3b shows that the ESR–temperature curves of the hydrogels with various CNT contents follow nearly the same variation law. Under low-temperature conditions, all the hydrogels show swelling behaviour. The equilibrium swelling ratio of the P-DN hydrogels was lower than that of single-network hydrogels (control group). The polymer chain density of the P-DN hydrogels is higher than that of single-network hydrogel, which hinders the swelling process of the materials. And the curve shows that, at 20 °C, compared with the P-DN hydrogel without the CNTs, the equilibrium swelling ratio of the P-DN hydrogel containing CNTs decreases by 6.5–13.6%. This can be attributed to the hydrophobic characteristics of the CNTs, which provide an explanation for the enhanced mechanical properties. When water molecules leave the hydrogel matrix, the equilibrium swelling ratio decreases and the polymer chain density increases, eventually leading to an increase in strength. At high temperatures, the equilibrium swelling ratio of all the hydrogels decreases considerably, which manifests as shrinkage due to water loss. In the range of 30 °C–35 °C, there is a transition from hydrophilicity to hydrophobicity. The temperature-response behaviour of the hydrogel is attributed to the PNIPAM component in the hydrogel. This transition means that PNIPAM is a typical LCST-type polymer. At low temperatures, the PNIPAM molecules form hydrogen bonds with water molecules and the macroscopic performance is water-absorption swelling. As the temperature increases, the hydrogen bonds between the water molecules and the PNIPAM molecules gradually break and most of the water molecules leave the interior of the hydrogel, which is reflected in the macroscopic shrinkage of the hydrogel.

### 3.3. Photothermal Conversion Test

**Figure 4:**
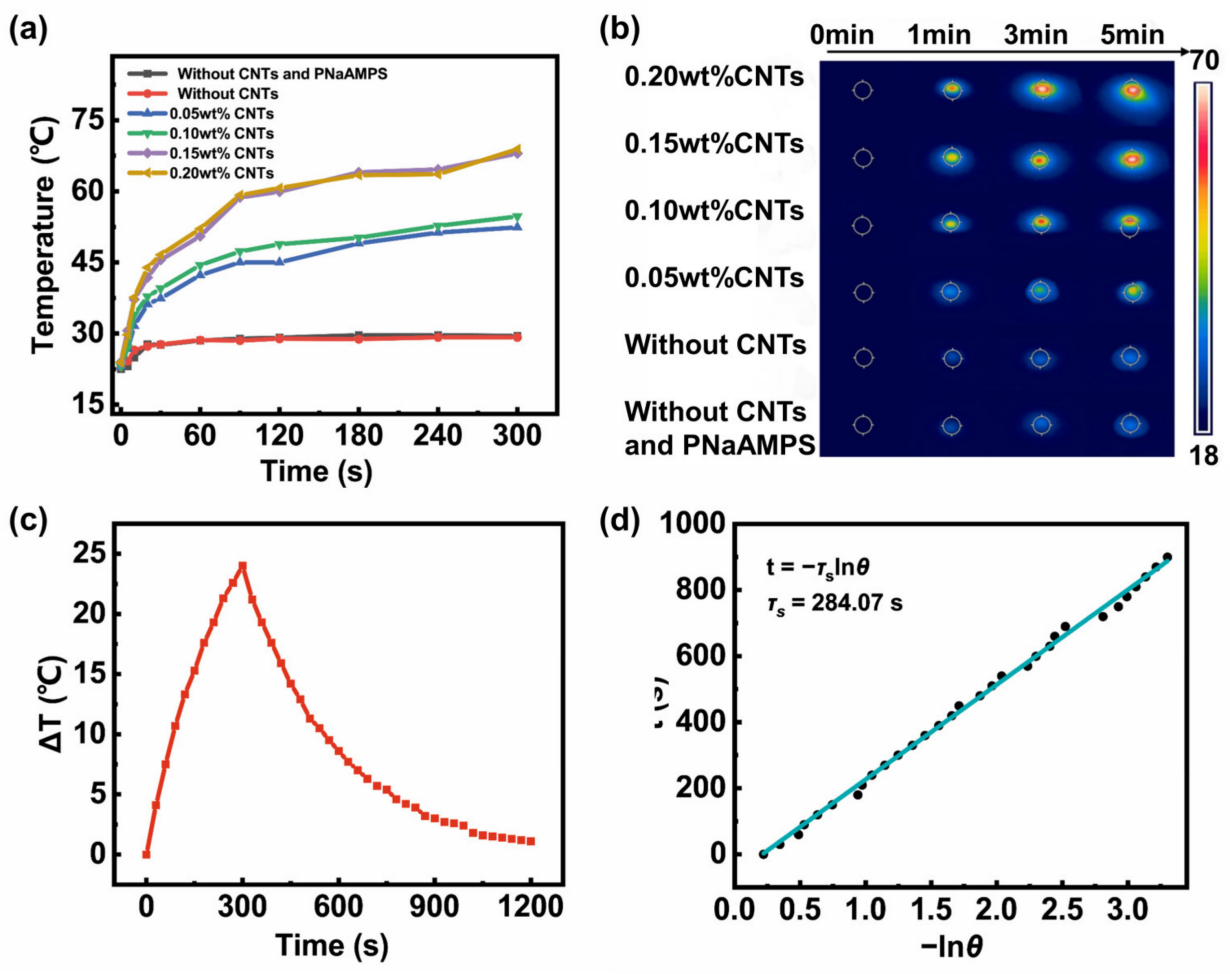
Photothermal conversion properties of hydrogels with PNaAMPS/P(NIPAM-co-MA)/CNTs, PNaAMPS/P(NIPAM-co-MA) and P(NIPAM-co-MA). (**a**) Temperature–time curves; (**b**) infrared thermal images of hydrogels under infrared light; (**c**) temperature change (ΔT) of the PNaAMPS/P(NIPAM-co-MA)/CNT hydrogel during NIR irradiation and NIR off; (**d**) linear time data versus the −ln(*θ*) fitting curves of the PNaAMPS/P(NIPAM-co-MA)/CNT hydrogel (obtained from the cooling period when the NIR laser was off).

Figure 4a shows that all the hydrogels with added CNTs exhibited photothermal conversion. In Figure 4b, each row represents the hydrogel sample exposed to near-infrared light at different irradiation times, while each column represents hydrogel samples with different CNT contents. The colour of the infrared images can vividly reflect the temperature of the samples. As shown on the temperature scale on the right, from colour blue to red, the temperature increases gradually. We could not detect an obvious change in temperature from the hydrogel without the CNTs; however, when the CNTs were introduced into the hydrogels, the temperature of the hydrogels was significantly higher than that of the surrounding environment, so it shows a red or yellow colour that is significantly different from background. And we can also observe that, as the content of the CNTs or NIR irradiation time increases, the pattern becomes larger and brighter, representing more heat emitted. With longer periods of NIR light irradiation, the surface temperature of the hydrogel with added CNTs rapidly rises. With the increase in the CNT content, the hydrogel surface temperature rise rate increases. This is attributed to the photothermal conversion effect of the CNTs. Compared with metal and inorganic materials, carbon-based materials, such as CNTs, have been selected as superior photothermal conversion materials because of their inherent excellent properties: they have extremely high light absorption in the vis and NIR bands, and a large number of conjugated six-membered carbon ring structures, which facilitate excited-state electron migration to transfer heat. In addition, because of the excellent thermal conductivity of the CNTs, heat can be quickly transferred to the hydrogel matrix. When the amount of CNTs added reaches 0.20 wt%, there is no significant improvement in the photothermal conversion performance. The possible reason is that, when the concentration of CNTs is high, the ability to absorb light tends to saturate (Figure S4). Therefore, we selected the sample with 0.20 wt% CNTs for subsequent research. Simultaneously, the hydrogel changes from transparent to opaque and exhibits a certain degree of water loss after being irradiated with near-infrared light, which also confirms the LCST properties of PNIPAM (Figure S5). Furthermore, we evaluated the recyclability of the photothermal conversion effect of the hydrogels. The hydrogel added with CNTs was irradiated under NIR for 5 min, and then the light was off to cool the hydrogel to near room temperature. The surface temperature of the hydrogel could still reach 64 °C after this process was repeated five times (Figure S6).

As a photothermal agent in hydrogels, photothermal conversion is a crucial characteristic. Therefore, the photothermal conversion performance of the carbon nanotubes was evaluated.

We dispersed CNTs in water and then irradiated them with a 980 nm NIR laser of 2 Wcm^−2^. We used pure water as the negative control group. As shown in Figure 4c, the temperature of the CNT solution rapidly increases under NIR radiation. After 300 s of irradiation, the temperature of the CNT solution was 53.9 °C (an increase of 24 °C). By contrast, the temperature of the pure water was 40.3 °C (an increase of only 10.4 °C). After 300 s of irradiation, the NIR laser was turned off and the reducing temperature was recorded for 900 s. The temperature change (ΔT) response to the NIR laser during 1200 s is shown in Figure 4c. Linear time data versus −ln (*θ*) was obtained from the cooling period of the NIR lasers. This relationship is shown in Figure 4b. The photothermal conversion efficiency of the CNTs (η) was calculated according to the following equation [31]:

$$\eta =\frac{hA\left(∆T_{max,mix}-∆T_{max,H_{2}O}\right)}{I\left(1-10^{-A_{\lambda }}\right)}$$

where *h* is the heat transfer coefficient, *A* is the surface area of the container, Δ*T_max_*_,*mix*_ and $∆T_{max,H_{2}O}$ are the temperature changes in the carbon nanotube aqueous dispersion and solvent (water), respectively, at the maximum steady-state temperature, *I* is the laser power and *A_λ_* is the absorbance of the carbon nanotube aqueous dispersion at 980 nm. For detailed information on the calculations, please refer to the reported literature [31,32]. According to this equation, we calculate the *η* of the CNTs to be approximately 21.73%.

### 3.4. Bending and Complex Deformation of Hydrogels

**Figure 5:**
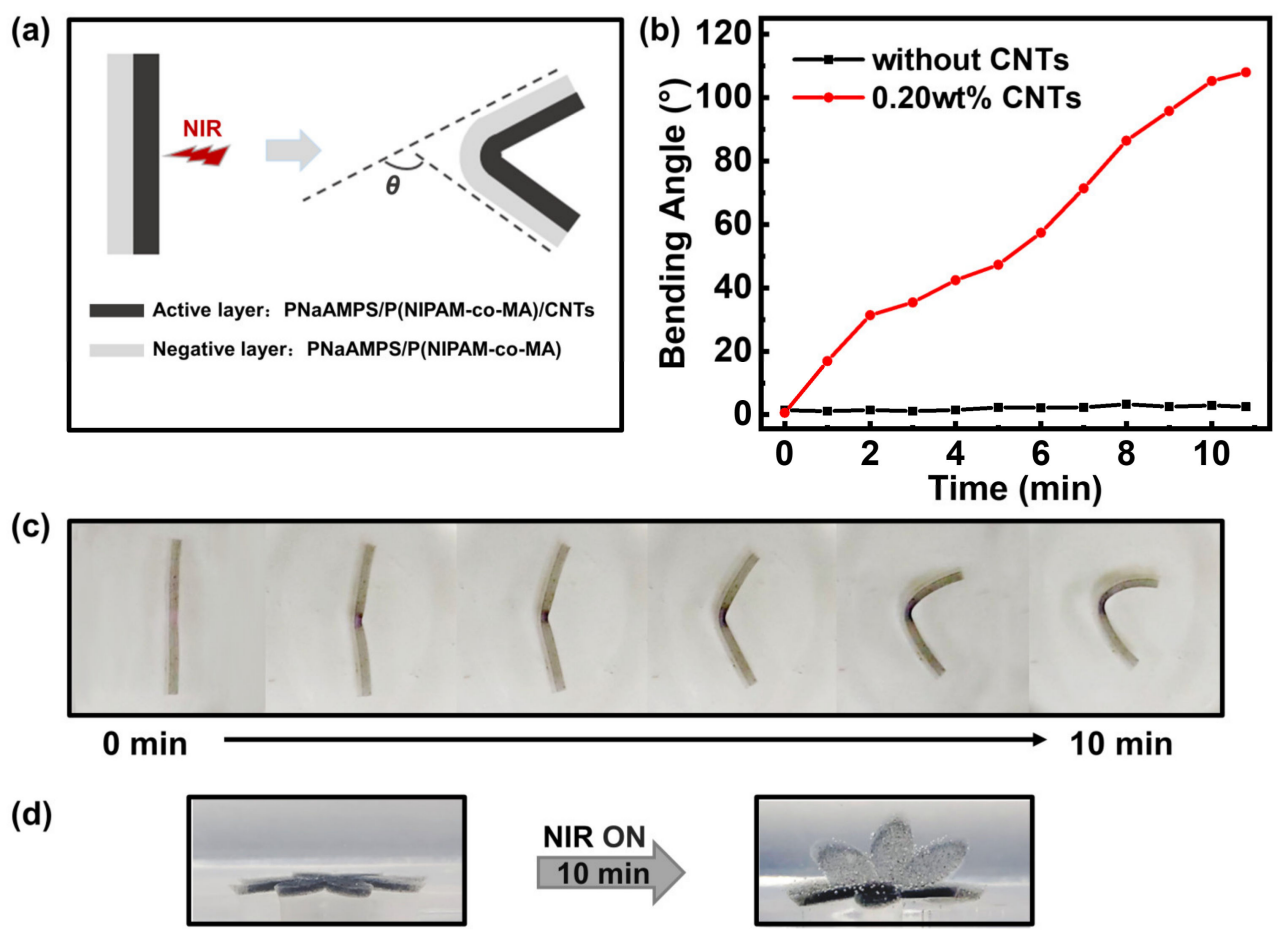
Deformation of the PNaAMPS/P(NIPAM-co-MA)/CNT hydrogel. (**a**) Schematic of the bending angle measurement, (**b**) bending angles of the hydrogels, (**c**) images of the bending angles and (**d**) images of the complex deformation.

To prepare the photoresponsive hydrogel actuators, we designed hydrogels with a bilayer structure. The structure of the hydrogel is shown in Figure 5a. The preparation method for the photoactive layer was the same as that used for the single-layer hydrogel. The second layer (negative layer) of the bilayer hydrogel does not contain CNTs, and the remaining steps are the same as those used for the first layer. When the NIR laser is on, the temperature of the active layer rapidly rises and the PNIPAM in the matrix undergoes phase transition and shrinkage, owing to dehydration. Owing to the shrinkage of the active layer and the swelling of the negative layer, the bilayer hydrogel bends towards the active layer. Using an infrared laser to illuminate the centre of the active-layer hydrogel, we recorded the changes in the hydrogel bending angles (as shown in Figure 5a) with the irradiation time. Figure 5b,c show that, when the control group (the bilayer hydrogel without CNTs) was irradiated, the bending angle of the hydrogel sample changed very little. When the bilayer hydrogel with 0.20 wt% CNTs was exposed to the NIR laser, obvious bending deformation was observed. The bending angles of the bilayer hydrogel (0.20 wt% CNTs in the active layer) rapidly increased from 0° to 110° within 10 min. This reflects the good photothermal conversion and deformation ability. The three-point bending test proves that the bilayer hydrogel has a sufficient bending modulus, which realises the design expectation (Figure S7). Through cyclic experiments, it was found that this deformation process can be repeated at least eight times (Figure S8). We cannot detect any creep phenomenon in such CNT-based bilayers during use. The introduction of the dispersed brittle PNaAMPS network of the microgels could serve as effective sacrificial bonds that yield and dissipate energy upon deformation. However, the destruction of such sacrificial bonds was permanent and could not be recovered. Meanwhile, the P(NIPAM-co-MA)/CNT copolymer molecular chain was covalently cross-linked by MBAA, and the broken covalent bonds in the system were also permanent and could not be recovered upon deformation.

Based on the bending deformation, we designed more complex three-dimensional deformation experiments, such as the folding and opening of flower-shaped hydrogels. The bilayer hydrogel was cut into complex geometric shapes (like a flower with eight petals) with a plastic mould and irradiated with an infrared laser, and deformation was observed, as shown in Figure 5d. The bilayer hydrogel changed from completely flat to slightly curved in about 5 min under infrared light irradiation and became completely folded like a flower after 10 min of continuous irradiation. After removing the infrared light for a certain period, the shape slowly recovered after deformation. We compared this work with the results of other authors [33,34,35,36,37,38,39] in Table S2. It is found that the hydrogel actuator both has reinforced mechanical properties and multiple stimuli-responsive functions, and can realise remote 3D deformation. So, it has unique advantages among many hydrogel drives.

## 4. Conclusions

Bilayer hydrogel actuators with adjustable mechanical properties, photothermal conversion ability and shape-deformation properties with fast response were prepared using a simple one-pot method. The construction of the microgel particle-enhanced double-network (P-DN) greatly improved the mechanical properties of the hydrogels, i.e., the dispersed brittle PNaAMPS network of the microgels can serve as sacrificial bonds to toughen the hydrogels. The mechanical properties of these materials could also be adjusted through photothermal conversion under NIR irradiation based on the LCST phase transformation of the PNIPAM molecular chains. By designing an asymmetric bilayer structure, the hydrogel actuators could display repeat deformation movement under NIR irradiation. Therefore, these P-DN hydrogels with multi-functionalities are attractive candidates for the remote control of soft robots and soft actuators.

## Figures and Tables

**Table 1:** Comparison of the mechanical properties of all hydrogel types.

Sample	σ (kPa)	ε (%)	W (kJ m^−3^)	E (kPa)
Without CNTs and PNaAMPS	45 ± 9	224 ± 29	65 ± 27	40 ± 9
Without CNTs	236 ± 16	324 ± 18	396 ± 39	47 ± 3
0.05 wt% CNTs	282 ± 11	405 ± 13	644 ± 21	77 ± 11
0.10 wt% CNTs	309 ± 19	357 ± 11	576 ± 14	73 ± 10
0.15 wt% CNTs	312 ± 20	335 ± 26	507 ± 25	79 ± 12
0.20 wt% CNTs	195 ± 7	310 ± 20	361 ± 37	69 ± 7

## Data Availability

Data are contained within the article and Supplementary Materials.

## Supplementary Materials

The following supporting information can be downloaded at: https://www.mdpi.com/article/10.3390/polym16081152/s1, Figure S1: The SEM images of original and modified CNTs (scale bar: 500 nm); Figure S2: The microstructural characterisation of the PNaAMPS/P(NIPAM-co-MA)/CNT hydrogel; Figure S3: The tensile stress–strain curve of the hydrogels (added with the original CNTs and modified CNTs); Figure S4: Transmittance curve of all hydrogels (PNaAMPS/P(NIPAM-co-MA), PNaAMPS/P(NIPAM-co-MA)/CNTs and SN: P(NIPAM-co-MA)); Figure S5: Optical photos of the phase-transition behaviour of the PNaAMPS/P(NIPAM-co-MA)/CNT hydrogels under near-infrared light irradiation; Figure S6: Cyclic reversible photothermal conversion behaviour of the PNaAMPS/P(NIPAM-co-MA)/CNT hydrogels (control group: PNaAMPS/P(NIPAM-co-MA) hydrogel); Figure S7: Three-point bending test of the PNaAMPS/P(NIPAM-co-MA)/CNT hydrogels (before and after NIR irradiation); Figure S8: Cyclic reversible bending behaviour of bilayer hydrogel (active layer: PNaAMPS/P(NIPAM-co-MA)/CNTs, negative layer: PNaAMPS/P(NIPAM-co-MA)) under infrared light irradiation; Table S1: The element ratio of the CNTs (original type and modified type); Table S2: Overall performance comparison of the PNIPAM-based hydrogel actuators.
