# Supplementary material for: NIR-Mediated Deformation from a CNT-Based Bilayer Hydrogel
Source: Polymers (Basel). 2024 Apr 19;16(8):1152. doi: 10.3390/polym16081152 (PMC11053785; doi:10.3390/polym16081152)
Supplement: Supplementary file 1 [file polymers-16-01152-s001.zip › polymers-2940203-supplementary.pdf]

Supporting Information for

NIR-mediated Deformation from a CNT-based Bilayer Hydrogel

Shijun Long[1,2,3*], Chang Liu[1], Han Ren[1], Yali Hu[1], Chao Chen[4*], Yiwan Huang[1,2] and Xuefeng Li[1,2,3*]

[1] *Hubei Provincial Key Laboratory of Green Materials for Light Industry, Hubei University of Technology, Wuhan, 430068, PR China*

[2] *Hubei Longzhong Laboratory, Xiangyang, 441000, PR China*

[3] *New Materials and Green Manufacturing Talent Introduction and Innovation Demonstration Base, Hubei University of Technology, Wuhan, 430068, PR China*

[4] *Hubei Key Laboratory of Polymer Materials, Hubei University, 430062, Wuhan, PR China*

[*]Corresponding author: longshijun.hp@163.com; chenchao@hubu.edu.cn; li_xf@mail.hbut.edu.cn

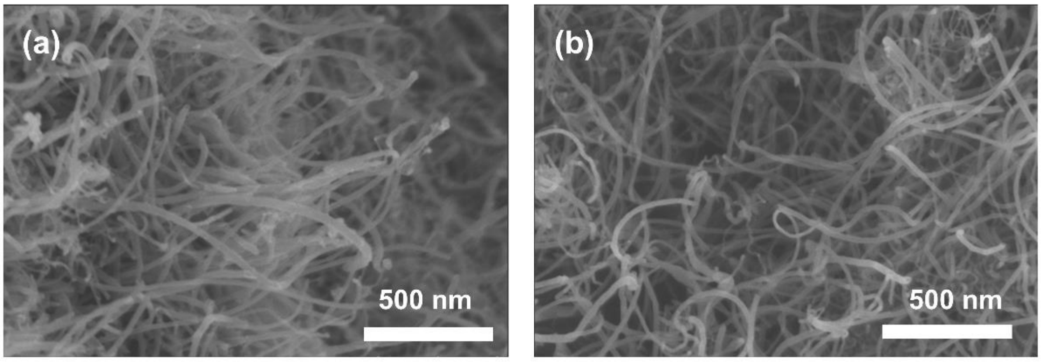

Figure S1. SEM photographs of original (a) and modified (b) CNTs. The scale bar is 500 nm

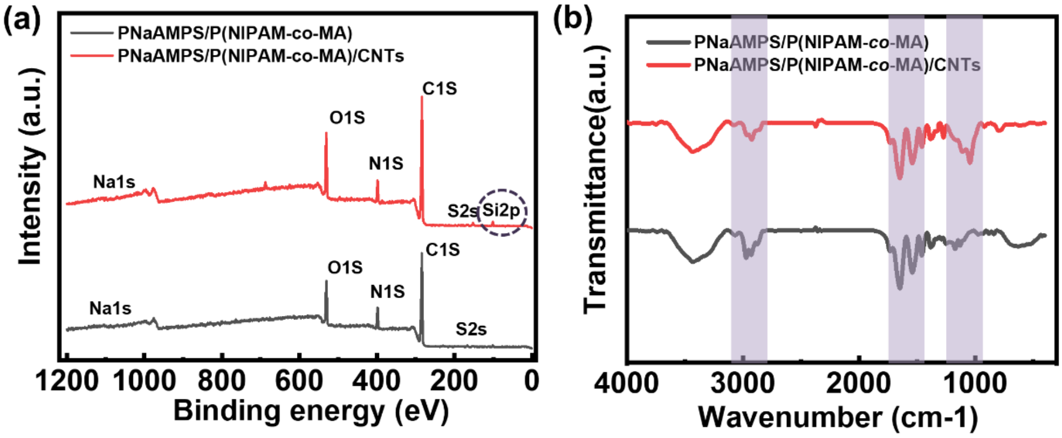

Figure S2. The microstructure characterization of dried PNaAMPS/P(NIPAM-co-MA) and PNaAMPS/P(NIPAM-co-MA)/CNTs hydrogel (CNTs content, 0.2 wt%). (a) XPS spectroscopy, (b) FTIR spectroscopy

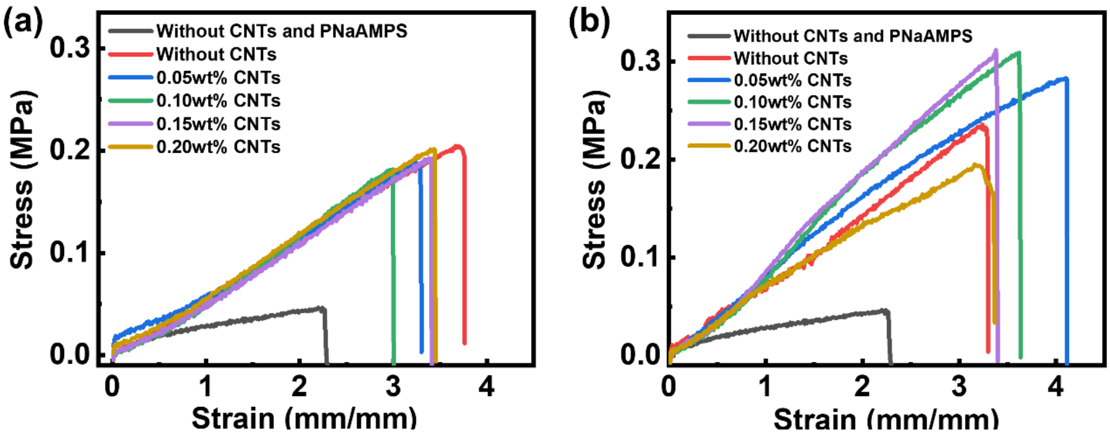

Figure S3. Tensile Stress-Strain curves of hydrogels(P(NIPAM-co-MA) hydrogel, PNaAMPS/P(NIPAM-co-MA) hydrogel and PNaAMPS/P(NIPAM-co-MA)/CNTs hydrogels) (a) hydrogels added with original CNTs (b) hydrogels added with modified CNTs

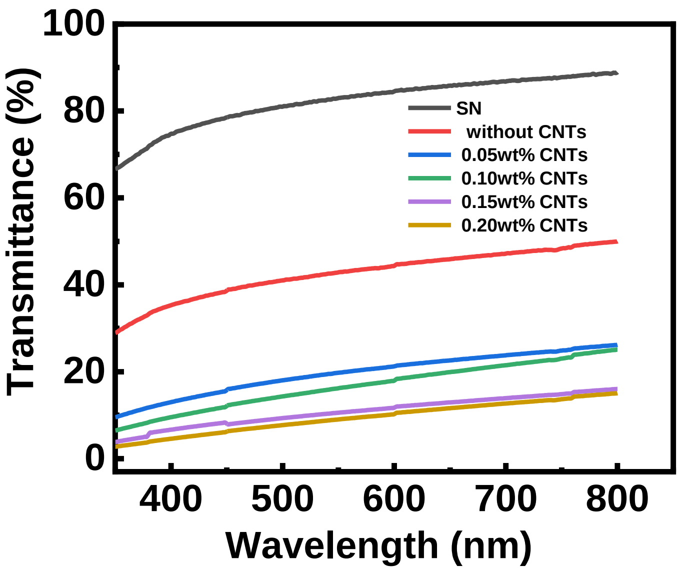

Figure S4. Transmittance curve of all hydrogels(PNaAMPS/P(NIPAM-co-MA), PNaAMPS/P(NIPAM-co-MA)/CNTs and SN: P(NIPAM-co-MA))

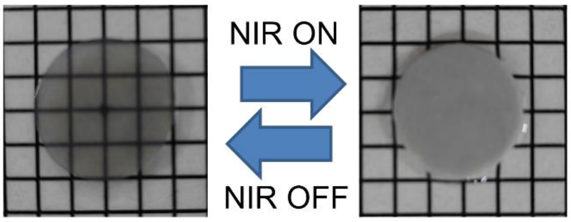

Figure S5. Optical photos of the phase transition behavior of PNaAMPS/P(NIPAM-co-MA)/CNTs hydrogels(CNTs content：0.20 wt%) under near-infrared light irradiation

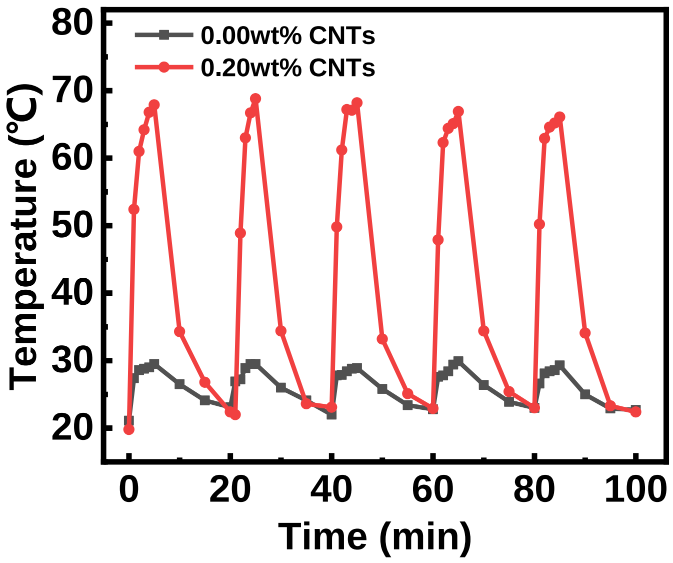

Figure S6. Cyclic reversible photothermal conversion behavior of PNaAMPS/P(NIPAM-co-MA)/CNTs hydrogels(Control group: PNaAMPS/P(NIPAM-co-MA) hydrogel)

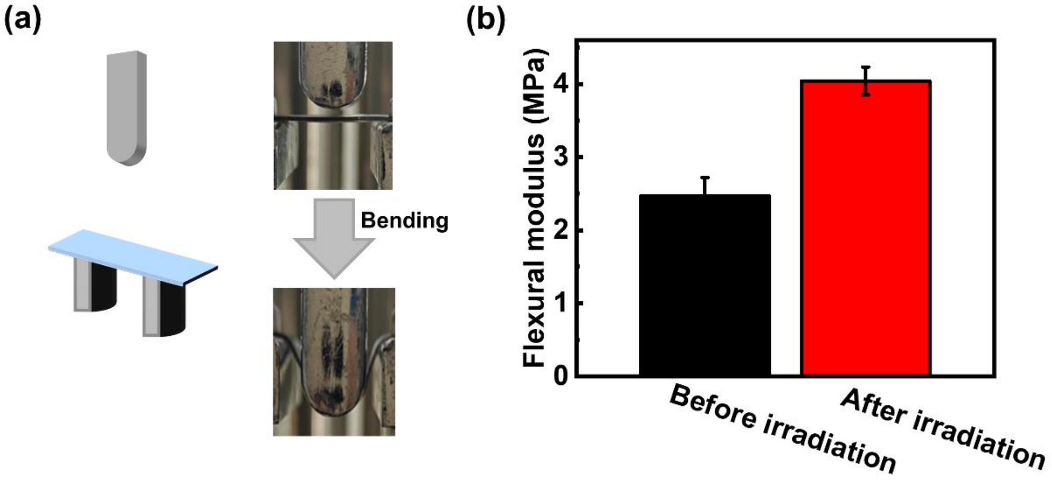

Figure S7. Three-point bending test of PNaAMPS/P(NIPAM-co-MA)/CNTs hydrogels (CNTs content, 0.2 wt%) before and after NIR irradiation. (a) Schematic diagram and optical images for three-point bending test; (b) Flexural modulus

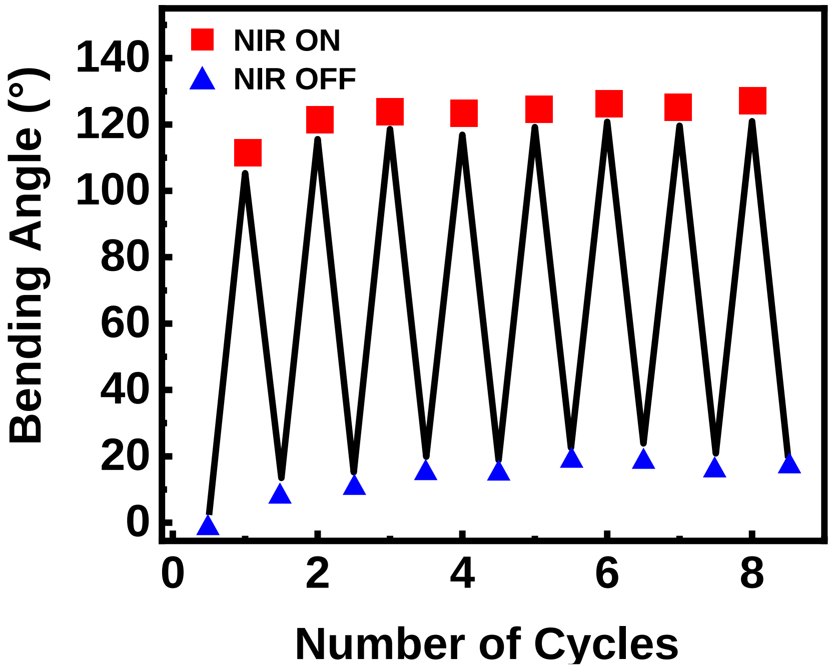

Figure S8. Cyclic reversible bending behavior of bilayer hydrogel(active layer: PNaAMPS/P(NIPAM-co-MA)/CNTs, negative layer: PNaAMPS/P(NIPAM-co-MA)) under infrared light irradiation

Table S1 The elements ratio of original and modified CNTs.

Elements	C	O	Si
Ratio(Original)	92.69%	7.21%	0.00%
Ratio(Modified)	86.24%	9.32%	4.44%

Table S2 Overall performance comparison of PNIPAM-based hydrogel actuators

Material	Structure	Stimuli-response type	Mechanic property	Deformation
This work	Bilayer	Temperature/light	Strengthen	3D
PNIPAM, Gelatin, PTCA, PAM[1]	Bilayer	Temperature/pH	No strengthen	3D
PNIPAM, PEDOT: PSS, Mxene[2]	Bilayer	Mechanic/light	No strengthen	3D
PNIPAM, rGO, ATPE, PLMA, 6APA[3]	Gradient	Temperature/light	No strengthen	3D
PNIPAM, Spiropyran[4]	Bilayer	Light	No strengthen	3D
PNIPAM, HEA, XLG[5]	Gradient	Temperature	No strengthen	2D
PNIPAM, Silicon Rubber[6]	Multilayer	Temperature	Strengthen	3D
PNIPAM, PAM, Nanothylakoid[7]	Bilayer	Temperature/light	No strengthen	3D

Reference of Table S2

[1] Wu, B. Y.; Le, X. X.; Jian, Y. K.; Lu, W.; Yang, Z. Y.; Zheng, Z. K.; Théato, P.; Zhang, J. W.; Zhang, A.; Chen, T. PH and Thermo Dual-Responsive Fluorescent Hydrogel Actuator. *Macromolecular Rapid Communications* **2018**, *40* (4), 1800648.

[2] Xue, P.; Valenzuela, C.; Ma, S. S.; Zhang, X.; Ma, J. Z.; Chen, Y. H.; Xu, X. H.; Wang, L. Highly Conductive MXene/PEDOT:PSS-Integrated Poly(N-Isopropylacrylamide) Hydrogels for Bioinspired Somatosensory Soft Actuators. *Advanced Functional Materials* **2023**, *33* (24), 2214867.

[3] Shang, H.; Le, X. X.; Si, M. Q.; Wu, S. S.; Peng, Y.; Shan, F. Q.; Wu, S.; Chen, T. Biomimetic Organohydrogel Actuator with High Response Speed and Synergistic Fluorescent Variation. *Chemical Engineering Journal* **2022**, *429*, 132290.

[4] Li, C.; Xue, Y. G.; Han, M. D.; Palmer, L. C.; Rogers, J. A.; Huang, Y. G.; Stupp, S. I. Synergistic Photoactuation of Bilayered Spiropyran Hydrogels for Predictable Origami-like Shape Change. *Matter* **2021**, *4* (4), 1377–1390.

[5] Tan, Y.; Wang, D.; Xu, H. X.; Yang, Y.; Wang, X. L.; Tian, F.; Xu, P. P.; An, W. L.; Zhao, X.; Xu, S. M. Rapid Recovery Hydrogel Actuators in Air with Bionic Large-Ranged Gradient Structure. *ACS Applied Materials & Interfaces* **2018**, *10* (46), 40125–40131.

[6] Visentin, F.; Murali, P.; Meder, F.; Mazzolai, B. Selective Stiffening in Soft Actuators by Triggered Phase Transition of Hydrogel-Filled Elastomers. *Advanced Functional Materials* **2021**, *31* (32), 2101121

[7] Zhao, H.; Huang, Y. M.; Lv, F. T.; Liu, L. B.; Gu, Q.; Wang, S. Biomimetic 4D-Printed Breathing Hydrogel Actuators by Nanothylakoid and Thermoresponsive Polymer Networks. *Advanced Functional Materials* **2021**, *31* (49), 2105544.
